# From sterile labs to rich VR: Immersive multisensory context critical for odors to induce motivated cleaning behavior

**DOI:** 10.3758/s13428-019-01341-y

**Published:** 2020-01-21

**Authors:** Jasper H. B. de Groot, Daniel J. V. Beetsma, Theo J. A. van Aerts, Elodie le Berre, David Gallagher, Emma Shaw, Henk Aarts, Monique A. M. Smeets

**Affiliations:** 1 Department of Psychology, Utrecht University, PO Box 80140, 3508 TC Utrecht, the Netherlands; 2 Department of Neurology, University of Pennsylvania, Philadelphia, PA USA; 3 Unilever R&D, Vlaardingen, the Netherlands; 4 Unilever R&D, Port Sunlight, UK

**Keywords:** VR, olfaction, motivation, behavior, sniff, context

## Abstract

Extending traditional research methods for studying the effects of odor on behavior, this study applied virtual reality (VR) to create a real-world, immersive context that was compared with a traditional sterile, non-immersive lab setting. Using precise odor administration with *olfactometry*, participants were exposed to three odors (cleaning-related pleasant smell, cleaning-unrelated pleasant smell: vanillin, and odorless air). Our aim was to tease apart whether participants’ motivation to clean was driven by cleaning associations and/or odor pleasantness, and how context would accentuate these effects. The results indeed showed that, in VR only, the cleaning-related smell elicited faster and more energetic cleaning behavior on a custom-designed cleaning task, and faster and more voluminous olfactory sampling compared with controls (vanillin, air). These effects were not driven by odor valence, given the general absence of significant differences between the pleasant control odor vanillin and odorless air. In sum, combining rigorous experimental control with high ecological validity, this research shows the context dependency of (congruent) odors affecting motivated behavior in an immersive context only.

**Electronic supplementary material:**

The online version of this article (10.3758/s13428-019-01341-y) contains supplementary material, which is available to authorized users.

## Introduction

Virtual reality (VR) offers a unique experimental tool to bridge the gap between theory and practice. VR has been applied to fields such as architecture, engineering, sales and marketing, education, therapy, and product testing. In psychological research, VR has the distinct advantage of immersing participants in realistic settings that remain under high experimental control, thus combining the strength of the lab (high internal validity) with the field (high external validity). Even though a VR setting becomes subjectively more realistic and immersive if multiple sensory channels are engaged (Josman, Reisberg, Weiss, Garcia-Palacios, & Hoffman, 2008), the application of VR has generally been limited to the visual domain and—to a lesser extent—hearing and touch, with the chemical senses (smell and taste) being overlooked (e.g., Carulli, Bordegoni, & Cugini, 2016).

VR has the potential for solving conflicting evidence in the chemical senses, by merging traditional lines of research performed in sterile lab environments versus in-home settings lacking rigorous control. Separate lines of research have shown that (i) *odors* can influence human perception, affect, and behavior (e.g., de Groot, Semin, & Smeets, 2017; Holland, Hendriks, & Aarts, 2005); (ii) odor perception can be modulated by *context* (e.g., de Araujo, Rolls, Velazco, Margot, & Cayeux, 2005); and (iii) the hardware exists to present smells in virtual environments with realistic precision (e.g., Dangelmaier & Blach, 2017). Yet, to our knowledge, VR has never been applied to dissect the contributions of context (real-world, immersive vs. non-immersive, sterile) and odor to changes in human perception and behavior. The present study provides a first test in the odor research domain and examines the effects of odor on behavior by applying VR and comparing this with traditional methods of odor research.

The human sense of smell is much better than traditionally thought. From the ancient Greeks, to Immanuel Kant and beyond, great thinkers have consistently called the human olfactory sense “inferior” (Le Guérer, 2002). This myth was fueled in the nineteenth century by Broca’s interpretation that our expanded frontal lobes and unique rationality allow humans to resist “animalistic” olfactory urges (reviewed in McGann, 2017). Although these pseudoscientific beliefs may have contributed to an initial scientific neglect of human olfaction, recent empirical studies have demonstrated excellent human smell skills. Like dogs, humans can track a scent trail through a field (Porter et al., 2007), and humans outperformed “super smellers” dogs and mice by detecting certain odorant molecules at lower concentrations (Can Güven & Laska, 2012). Odors serve various important functions in humans, from judging the edibility of food, to escaping noxious gases (Stevenson, 2010), and social communication (de Groot et al., 2017; Parma, Gordon, Cecchetto, Cavazzana, & Lundström, 2017; Pause, 2017; Stevenson, 2010). Hence, countering pervasive fictional views, these studies have highlighted functional and exceptional human olfaction.

The importance of smells in our everyday lives has been realized by the consumer product industry, which devotes billions of dollars each year to determining which fragrance best enhances so-called fast-moving consumer goods such as perfumes, foods, cosmetics, and home care products (Hoover, 2010). The lion’s share of product testing is based on long-standing sensory practices derived from experimental psychology, with trained panelists rating smells in cubicles that are deprived of other sensory input (Lawless & Heymann, 2010). The alternative is in-home product testing, which—despite high ecological validity—is less popular due to its high costs and poor experimental control. The present dichotomy (lab vs. field) in sensory testing is problematic and requires an integrated approach combining the strengths of the lab (control) and the field (ecological validity), because there are strong reasons to assume that responses to odors in sterile labs are not predictive of responses to odors in naturalistic settings.

Indeed, prior research has demonstrated that the perception of certain odors could be changed (even inverted) at the hedonic level as a function of the verbal context in which the odor was presented. When labeled “parmesan cheese”, a mixture of isovaleric and butyric acid (I-B acid) was well liked, whereas I-B acid was detested when labeled “vomit” (Herz & Von Clef, 2001). Another example is menthol, which was found pleasant when labeled “breath mint”, but unpleasant when labeled “chest medicine” (Herz & Von Clef, 2001). In a functional magnetic resonance imaging (fMRI) study, the same smell (isovaleric acid) also yielded different patterns of brain activity (anterior cingulate cortex, medial orbitofrontal cortex), depending on the label that was tied to the smell (“body odor” vs. “cheddar cheese”) (De Araujo et al., 2005). When participants were led to believe that an odor was “harmful” (vs. “healthful”), the same odor even led to more reported health symptoms (Dalton, 1999); these *expectation effects* already impact odor perception at the earliest processing levels (Bulsing, Smeets, Hummel, & van den Hout, 2007; Laudien, Wencker, Ferstl, & Pause, 2008). Together, these studies have shown that higher-order cognitive input is crucial to odor experience, and the poor ecological validity of traditional odor testing performed in sterile labs could be considered a concern. For this reason, we introduced VR to create an enriched, yet controlled, “real-life” context that involved multisensory stimulation (vision, hearing, touch, and smell), and we contrasted this context with odor experience in a non-immersive condition that resembled a more traditional lab setting.

Even though research has already shown that odor *perception* can be altered by context, to date no research has examined whether the context can also change *motivated behavior* following exposure to smells. Prior research has shown that motivated behavior can be induced by certain odors, usually without our awareness. Compared with a no-scent condition, participants that were inconspicuously exposed to a cleaning agent (citrus) smell (i) were more likely to have spontaneous thoughts about cleaning, and (ii) were shown to clean more crumbs from their desk after eating a rusk (actual cleaning behavior) (Holland et al., 2005). These findings were replicated in a field study (de Lange, Debets, Ruitenburg, & Holland, 2012), which showed that passengers littered less in a citrus-scented train wagon than in an unscented wagon.

It is important to note that these studies measured just the *endpoint* of cleaning behavior, either in the lab or in the field, which substantially limits the understanding and application of odor effects on behavior. First, we cannot make inferences about actual *motivated* cleaning behavior, because effects were not assessed on specific measures that typify motivated behavior, such as force and speed of action (Aarts, Custers, & Marien, 2008). Second, we cannot dissect the *specific* contribution of the test context to motivated behavior. Third, because previous studies lacked an odorous control condition for citrus smell, alternative explanations related to the valence of the odor cannot be ruled out. For instance, odors equally pleasant to citrus could have induced the same behavior, as it is known that positive affect can (implicitly) motivate behavior (e.g., Custers & Aarts, 2005). Fourth, odor delivery was not tightly controlled in previous studies, so we cannot be sure that the presented odors actually reached participants’ noses and were inhaled, potentially dampening the studies’ effect sizes. Our aim is to supplement the existing literature by accounting for these remaining issues in the present research.

## Present research

Here, we adopted *VR* to create a realistic, immersive, yet controlled multisensory context (that was contrasted with a non-immersive, traditional lab setting), and we applied *olfactometry* to ensure precise, realistic, computer-controlled odor delivery. This setup allowed us to combine the strengths of lab and field research. So far, only a few attempts have been made to integrate odors into VR using an olfactometer, and these reports focused mostly on VR functionality, namely how smells would enhance subjective “presence” in VR (e.g., Ariyakul & Nakamoto, 2011; Bordegoni & Carulli, 2016; Carulli et al., 2016; Dhokia et al., 2016; Howell, Herrera, Moore, & McMahan, 2016; Porcherot et al., 2018); far fewer studies have applied VR to study the way smells influence our perception and behavior (Discalfani, 2012; Li & Bailenson, 2018; Quintana, Nolet, Baus, & Bouchard, 2019).

Our aim was to tease apart the specific contributions of certain *odors* and *context* to perception and motivated behavior. Based on gaps in the previous literature (de Lange et al., 2012; Holland et al., 2005), we exposed participants to *three* odors: (i) a pre-validated cleaning-related odor (laundry odor), (ii) vanillin (iso-pleasant control odor, semantically unrelated to cleaning), and (iii) room air (odorless control). Based on previous research (de Lange et al., 2012; Holland et al., 2005), we expected (i) a cleaning-related smell (vs. odorless control) to induce a greater motivation to clean. Second, we explored whether (ii) an equally pleasant control odor (vanillin) could enhance participants’ motivation to clean (vs. odorless control), based on research showing the motivating properties of positive affect (Custers & Aarts, 2005). Crucially, participants perceived the three odors in either an immersive washing-related VR setting or in a non-immersive two-dimensional (2D) condition resembling a traditional lab setting. We expected any effect(s) of odor to be (iii) *more pronounced* in an immersive VR setting versus a non-immersive 2D “lab context”, because the immersive washing-related context has an expected greater ability to fuel motivation to clean through realistic multisensory stimulation (Barsalou, 2009, 2016b).

In our lab, we custom-designed a cleaning task (participants had to manually remove a stain from a piece of clothing) that formed a logical follow-up to the washing scenario that was part of the context (VR, 2D) manipulation. Motivation to clean was assessed with behavioral features that are typical for motivation, namely participants’ hand movements (hand force, hand speed, total time spent on cleaning task) that were recorded with an unobtrusive logging device. We also measured odor sampling (intranasal cannula), to explore whether the degree of odor intake in VR/2D (sniffing) could be modulated by odor and context.

## Method

The Ethics Committee of the Faculty of Social and Behavioral Sciences of Utrecht University (Utrecht, the Netherlands) approved this research (FETC15-094).

### Participants and design

To compute minimum sample size, we used G*Power (Faul, Erdfelder, Lang, & Buchner, 2007), which suggested *N* = 84, based on a mixed analysis of variance (ANOVA) with two groups and three measurements, a low (0.2) repeated-measures correlation, 90% power, α = .05, and the lowest effect size (η_*p*_^2^ = .08; vs. .15, .23) reported in best matching prior research (de Lange et al., 2012; Holland et al., 2005). Anticipating dropouts/missing data, we tested 90 participants.

Ninety healthy, non-smoking female undergraduates (*M*_age_ = 22.14 years; *SD*_age_ = 4.96 years) provided written informed consent to participate in this experiment in return for course credit or €8. Only female participants were recruited, because their generally better olfaction (Sorokowski et al., 2019) makes them the most sensitive sample to test our proof of principle. Non-inclusion occurred in the case of self-reported mental or physical illness, daily medication usage, abnormalities in the sense of smell, respiratory diseases, allergies, a current cold, sickness, pregnancy, or being a Unilever/Utrecht University employee.

Participants enrolled in a 3 × 2 mixed design, with odor (3 levels: laundry odor, vanillin, air) manipulated within subjects, and context (2 levels: VR, 2D) between subjects. Participants were equally distributed over the VR (*M*_age_ = 22.11 years, *SD*_age_ = 4.68 years) and 2D context (*M* = 22.18, *SD* = 5.27). Odor presentation was counterbalanced.

### Materials and measures

#### Odors

Next to room air (odorless control), the odor stimuli were a pre-validated laundry odor and vanillin (4-hydroxy-3-methoxybenzaldehyde; supplier: Sigma-Aldrich). These odors and their concentration levels (laundry odor: 0.8% volume percentage in odorless propylene glycol; vanillin: 10% weight percentage in propylene glycol) were selected based on several pilot tests (*N*_total_ = 35). The goal of these pilot tests was to select two equally intense and equally pleasant odors, of which one (and not the other) would activate washing- and cleaning-related concepts. The pilot tests revealed that ratings of 0.8% laundry odor and 10% vanillin had considerable overlap in terms of their intensity (laundry: *M* = 6.28, *SD* = 1.09; 95% CI: 5.56–6.99; vanillin: *M* = 5.20, *SD* = 2.56; 95% CI: 3.53–6.87) and pleasantness (laundry: *M* = 6.86, *SD* = 1.50; 95% CI: 5.87–7.84; vanillin: *M* = 6.25, *SD* = 0.89; 95% CI: 5.67–6.83). Furthermore, laundry odor was described as “detergent”, “soapy”, and “washing powder”, whereas vanillin was not at all cleaning-related: “vanillin, sweet vanilla ice-cream”, and “something sweet, perhaps edible”.

#### Odor delivery

To present odors (and air) to participants in a software-controlled manner, a triple-channel airborne olfactometer was custom-built at Utrecht University out of stainless steel and Teflon components, minimizing cross-condition odor contamination. Of the three separate evaporation chambers, one was empty (odorless control), whereas two contained a small glass petri dish (height: 0.5 cm; ø: 5.8 cm) with 3 ml of either laundry odor or vanillin. The triple-channel design was maintained throughout the olfactometer, from the evaporation chambers to the tubes delivering the odor to approximately 2 cm from the participant’s nose. The delivery end of the olfactometer was connected to the VR glasses and chin rest (2D context), with three separate *silicon* tubes to allow for natural head movements. For each odor channel, a set of Teflon valves directed the odorized airflow (4 l/min) to the participant’s nose, or toward a ceiling air suction device. This enabled an “odor preparation loop” that was activated 30 s prior to odor release, to ensure a discrete stimulus onset (i.e. no intensity buildup). The air input pressure of the lab was 8 bar, then reduced to the required 4 bar for three flow controllers. A 30 mbar Teflon check valve was placed in each channel for safety purposes. The olfactometer was software-controlled by a custom software package written in LabVIEW (National Instruments, Austin, TX).

#### Context

The goal of this manipulation was to create an immersive, realistic, enriched VR laundry context that (paired with laundry odor) would increase motivation to clean, compared with a similar but non-immersive 2D version.

**Fig. 1:**
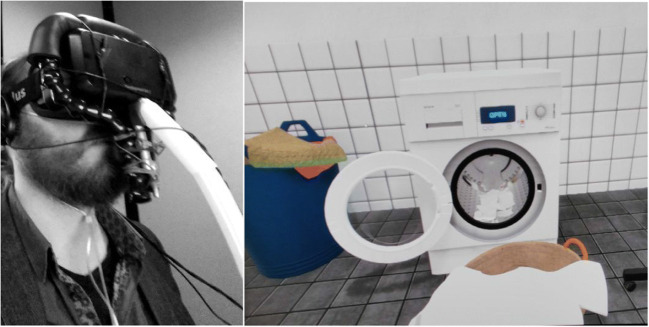
A view of the olfactometer connected to the Oculus Rift (left), and what a participant sees from within the VR environment (right)

The VR scenario was designed by CleVR B.V. (Delft, the Netherlands), specialized in creating customized VR scenarios. The scenario entailed a laundry environment with a spinning washing machine programmed in Unity, which was presented using Oculus Rift DK2 3D glasses and headphones. Participants were virtually seated in front of the washing machine, which was spinning audibly. When the washing machine ended its cycle, participants had to press a controller to open the washing machine door, after which they saw a t-shirt (with a just noticeable stain) falling out of the washing machine that landed in a laundry basket. The stain was pilot-tested to be just noticeable to create a *possibility* that participants would be motivated to manually clean the t-shirt in a subsequent cleaning task (e.g., when laundry odor was embedded in VR). To present odors while participants experienced the virtual world, the Oculus Rift was modified to hold the delivery end of the olfactometer (Fig. 1).

The 2D context entailed a computer screen showing a cropped screenshot of the VR scenario with the stained t-shirt lying in the laundry basket. This mode of presentation is more representative of traditional non-immersive sensory testing excluding multisensory stimulation (sound, touch) and movement. The image was presented using E-prime 2 stimulus presentation software (17” monitor; 1280 × 1024).

#### Sniffing responses

Sniffing responses/breathing were monitored for two reasons: (i) to ensure that participants took in the odors, and (ii) to explore whether different conditions (odor, test context) would induce different patterns of air intake (cf. de Groot, Smeets, Kaldewaij, Duijndam, & Semin, 2012). Continuous tracking of nasal air pressure (with changes reflecting sniffing/breathing) was achieved with high temporal resolution (Johnson, Russell, Khan, & Sobel, 2006) using a silicon nasal cannula (SleepSense) that was inserted ~0.5 cm into the participant’s nose. Changes in air pressure were A/D-converted by a calibrated multi-press pressure transducer (SleepSense), before being time-synchronized with the olfactometer by a Biopac MP150 data acquisition system and AcqKnowledge software (Biopac Systems, Goleta, CA), with digital triggers indicating the exact moment of odor release. Because of this, pre-stimulus baseline breathing (−30 to 0 s) could be neatly separated from post-stimulus sniffing responses (0 to 15 s). Offline, data were low-pass finite impulse response (FIR)-filtered (16 Hz) using custom-developed software (Sniff Analyzer, Utrecht University, Utrecht), and inhalations were identified based on any negative air pressure ≥ 0.4 s in length, *and* any negative air pressure ≥ 10% of the largest post-stimulus pressure per subject, per condition (de Groot et al., 2012). Inhalations overlapping with odor onset were considered invalid, except when ≥ 80% of its area under the curve lay pre- *or* post-stimulus, in which case the inhalation was classified as pre-stimulus *or* post-stimulus, respectively.

#### Cleaning task

**Fig. 2:**
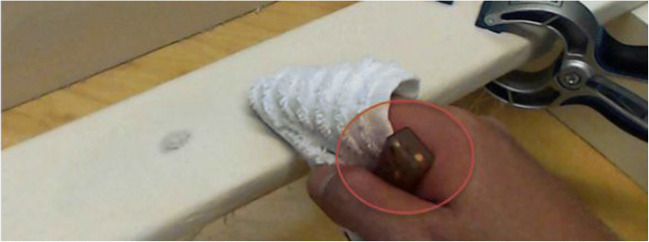
Display of the cleaning task: the plank with stained linen, the kitchen rag, and the movement logger (red circle)

The aim of this task was to measure participants’ motivation to clean. After being exposed to odor and context, participants were instructed to rub a stain out of a piece of unbleached natural linen. The linen cloth was attached via spring clamps to a wooden beam, which was fixed on a wooden construction on a desk to prevent the plank from moving and to ensure participants could comfortably perform the rubbing task. Each cloth contained a stain (2 cm ø) that was created using an HB pencil (Fig. 2). Pilot tests had shown that this stain could be fully removed within 10–30 s, depending on a person’s motivation to clean. To clean the stain, participants were given a kitchen rag and a bowl of water (~30 ml) with one drop of fragrance-free detergent. The three bowls of liquid (one per odor condition) were labeled A, B, and C, and participants were told that each bowl contained a different cleaning liquid, with the aim of the cleaning task being to identify the most effective cleaning liquid. Participants had to wrap a corner of the kitchen rag around their finger, dip this corner into the cleaning liquid, and clean the fabric until they decided the task was finished. Meanwhile, cleaning behavior was recorded by webcam and small acceleration loggers (45 × 17 × 12 mm), which measured acceleration on three axes (*x*, *y*, *z*) with a sampling frequency of 20 Hz (measurement range: ± 8 g). Without revealing the purpose of the logger, participants were instructed to hold a device in the palm of their hand while they performed the cleaning task. Data thus collected were filtered with a 1 Hz high pass for each axis, and combined (by hypotenuse) to arrive at net acceleration. Additional outcome variables emerged from calculating the auto power spectrum (1–10 Hz) of this filtered signal (see Statistical analysis).

#### Subjective measures

Participants also completed questions on visual analog scale (VAS) items that served as manipulation checks (see Supplementary results).

Regarding odor, participants indicated perceived odor pleasantness (0 = “not pleasant at all”, 100 = “very pleasant”), and intensity (0 = “I do not smell anything”, 100 = “I can smell the odor very clearly”). They also associated each odor with a certain activity (0 = “not at all”, 100 = “very much”), such as laundry (e.g., hanging up the laundry), dishes (e.g., washing pans), or food (e.g., cutting a cake).

Regarding context, participants in VR answered five Likert-scale questions about immersion and realism on seven-point Likert scales (1: “not at all”; 7: “very much”; e.g., “to what extent did the experiences in VR match the real world?”). Participants in VR and 2D were asked after each odor exposure to judge the cleanliness of the t-shirt that fell out of the washing machine (0 = “not … at all”, 100 = “very …”; e.g., “how white/dirty/clean do you think the t-shirt is?”), as potential differences in perceived t-shirt cleanliness could affect subsequent washing task performance.

Finally, participants were asked about their laundry habits in daily life, including the frequency of doing (manual) laundry.

### Procedure

**Fig 3:**
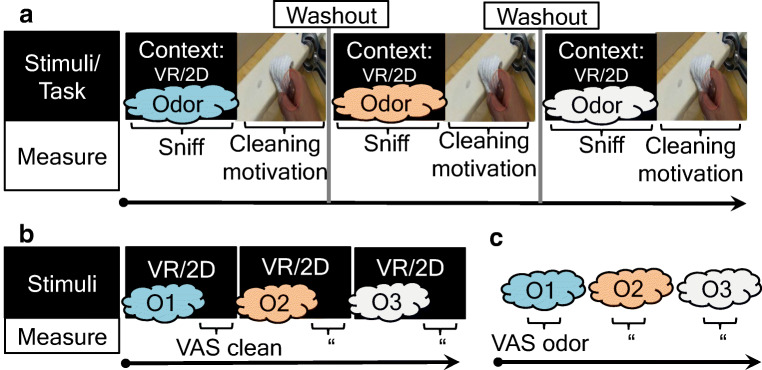
Study timeline showing experimental manipulations, task, and recordings. Context: Immersive VR *or* non-immersive 2D (between subjects); all participants smelled three counterbalanced odors (laundry, vanillin, odorless control). **a** Main experiment. **b, c** Subsequent manipulation checks

The experimental procedure is outlined in Fig. 3. In Block A, participants were exposed to one of three odors (counterbalanced) in a particular context (VR vs. 2D), while sniffing responses were recorded. Afterward, they performed the cleaning task. This sequence was twice repeated, once for each remaining odor. Explicit manipulation checks (Block B, C: details below) were administered after Block A to prevent odor or t-shirt stain awareness from affecting implicit sniffing and cleaning responses. The experiment ended with questions regarding the participants’ washing habits, immersion (VR only), and their awareness of the study’s hypothesis, after which they were debriefed, thanked, and paid.

#### VR procedure

Participants were equipped with a nasal cannula, headphones, and VR glasses with integrated olfactometer. They were instructed to hold an X-box controller. Participants were familiarized with VR in a standard Oculus Rift test environment to fine-tune visual settings and to screen for motion sickness. Then, Block A started. Participants were instructed that they would virtually enter a bathroom in which a washing machine had nearly finished a cycle (30 s), and that it would beep when it was done, after which they would have to “manually” open the door using a button on the controller. The exact moment of door opening triggered (i) a t-shirt (with stain) falling into the laundry basket, and (ii) odor release. After 15 s, the VR scenario and odor presentation ended, and participants performed the cleaning task, using the cleaning liquid in bowl A. This sequence was twice repeated to include different odor primes and cleaning liquids (bowl B, C). After the third cleaning task, participants were asked which liquid best removed the stain.

In Block B, the cleaning task was replaced by questions (VAS items) that scored participants’ perceptual evaluation of the t-shirt that left the washing machine. Participants again had to open the washing machine door, and while odor presentation stopped after 15 seconds (cf. Block A), they remained in VR to answer VAS items that appeared on a virtual screen (to the right of the washing machine) using the controller. Again, this sequence was twice repeated to present all odors. The same went for Block C, in which pleasantness and intensity ratings of all three odors were collected ex situ, using just the olfactometer.

#### 2D procedure

Participants placed their heads in a height-adjustable chin rest, which held the olfactometer tube that was aimed at the nostrils at ~2 cm. The nasal cannula was attached, and sniffing recording started. Participants looked at a black screen that displayed the text “please wait … the experiment will continue shortly” for 30 s (analogous to participants in VR viewing the spinning washing machine for 30 s). After 30 s, odor release coincided with a still picture from the VR scenario of the t-shirt containing a just noticeable stain. Analogous to VR, this stage lasted for 15 s, after which odor presentation stopped and the screen turned black. Participants then performed the cleaning task (identical to VR). This sequence was twice repeated, until all odors were presented. The nasal cannula was then removed. In Block B, participants answered the VAS items that appeared on a large (30”) monitor to the right of the participant, using the hand controller. Block C was identical to VR.

### Statistical analysis

#### Sniffing data preparation

**Fig. 4:**
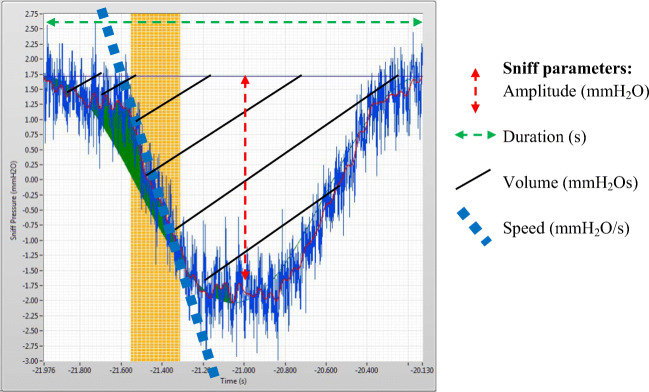
Example recording of sniff: change in nasal air pressure (cf. legend, for parameters)

As sniffing responses were expected to deflect from regular breathing, sniffing responses were calculated (and standardized) by subtracting per participant per condition their pre-stimulus baseline from post-stimulus air intake. The final six pre-stimulus in-breaths served as the baseline, whereas the first three sniffs post-stimulus charted the modulation of air intake as a function of our experimental manipulations (see pre-registration). Each sniff contained information about amplitude (maximum observed pressure: mmH_2_O), duration (sniff length: s), area under the curve (total inhalation volume: mmH_2_Os), and sniff speed (Fig. 4). Sniff speed (i.e., the maximum downward slope of the air intake acceleration: mmH_2_O/s) was added based on the notion that sniffing usually entails an (audible) increase in inhalation velocity compared with the standard respiratory pattern, and this increased velocity has been linked to enhanced odor perception (Laing, 1983; Mainland & Sobel, 2006).

#### Cleaning task data preparation

Behaviors on the cleaning task were recorded with a webcam, and these video data and acceleration logger data were examined together to select “washing blocks”: time segments during which the participant was manually cleaning the stain. This was done using a custom-written LabVIEW acceleration analysis program. All washing blocks were then combined to calculate several outcome variables to chart motivation to clean. Whereas *total cleaning time* was simply the total time participants spent cleaning, a *time-standardized* measure of *total cleaning effort* was obtained by dividing the area under the curve (AUC) in the acceleration × time domain (m/s) by total cleaning time, with higher values meaning more invested effort per second (i.e., more cleaning motivation). The auto power spectrum was used to calculate *typical hand movement frequency* (i.e., the peak/mode of all hand movement frequencies in Hz) and other variables that were tailored toward charting cleaning strategy rather than invested energy (see Supplementary results).

#### Statistical analysis

Outliers: For cleaning task parameters (not standardized), outliers were identified with the most robust scale measure in the presence of outliers, namely values surpassing three median absolute deviation (MAD) units (Leys, Ley, Klein, Bernard, & Licata, 2013). When outliers were identified, their values were changed to be one unit on that variable’s scale above the next extreme score on that variable that was not an outlier (Field, 2009, p. 153; Field, 2013, p. 198), a procedure also used in our previous research (e.g., Kamiloglu, Smeets, de Groot, & Semin, 2018; van Nieuwenburg, de Groot, & Smeets, 2019). As sniffing data were standardized per subject and per odor, outlier analysis was deemed unnecessary. Missing data: 2.6% of cleaning task data were lost due to acceleration logger recording errors, while missing sniffing data included recording errors (1.1%), missing odorless control baseline data (1.1%), and participants not producing three sniffs fitting our minimum criteria (laundry odor: 5.6%; vanillin: 7.9%; air: 6.7%).

## Results

### Motivation to clean

First, we tested how different odors and contexts would impact participants’ motivation to clean on a custom-designed cleaning task. Enhanced cleaning motivation was expected to follow exposure to laundry odor (vs. vanillin, air) in an immersive VR context (vs. non-immersive 2D). Cleaning motivation was inferred from three parameters derived from hand movement logger and video recordings: (i) standardized total cleaning effort, (ii) time to finish cleaning, and (iii) typical hand movement frequency (for additional redundant parameters, see Supplementary results).

#### Standardized total cleaning effort

**Fig. 5:**
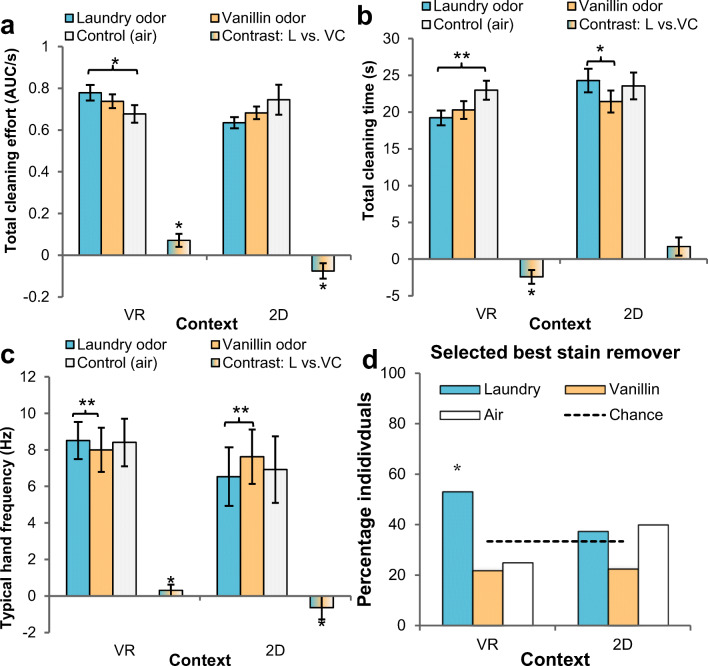
Mean cleaning behavior (**a**: total cleaning effort; **b**: total cleaning time; **c**: typical hand movement frequency) and perception of effective cleaning (**d**: percentage of participants choosing cleaning liquid from particular condition as best stain remover) as a function of odor (within subjects: laundry, vanillin, air) and context (between subjects: VR, 2D). For cleaning behavior variables (**a–c**), multicolored bars show within each context (VR, 2D) the planned contrast of laundry odor versus both control odor (vanillin) and odorless control (air). Error bars ± 1 SE. VR = virtual reality condition; 2D = two-dimensional condition. “Contrast L vs. VC”: contrast laundry odor vs. vanillin and odorless control. *p < .05, **p < .01

Total cleaning effort was obtained by dividing the area under the curve (AUC) in the acceleration × time domain (m/s) by the time participants took to complete the task. A repeated-measures (RM) ANOVA with within-subjects factor odor (3 levels: laundry, vanillin, air) and between-subjects factor context (2 levels: VR, 2D) showed a significant odor x context interaction, *F*(2, 170) = 4.27, *p* = .016, η_*p*_^2^ = .05 (odor: *F* < 1; context: *F* < 1). Planned contrasts then compared within each context (VR, 2D) whether laundry odor (vs. vanillin and odorless control) affected total cleaning effort. Indeed, when laundry odor was embedded in VR, total cleaning effort was significantly higher (vs. both controls), *F*(1, 42) = 5.22, *p* = .027, η_*p*_^2^ = .11 (laundry vs. air, *F*(1, 42) = 4.97, *p* = .031, η_*p*_^2^ = .11; laundry vs. vanillin: *F*(1, 42) = 2.08, *p* = .156, η_*p*_^2^ = .05), with the 2D context yielding a different (inverted) pattern, *F*(1, 42) = 4.36, *p* = .043, η_*p*_^2^ = .09 (Fig. 5a). In VR, a pleasant odor per se was incapable of increasing cleaning effort (vanillin vs. air), *F*(1, 42) = 1.97, *p* = .167.

#### Total cleaning time

Next, we looked at the time it took participants to finish the cleaning task. Total cleaning time was inversely correlated with total cleaning effort (laundry: Spearman’s ρ(87) = −.33, *p* = .002; vanillin: ρ(86) = −.36, *p* < .001; air: ρ(87) = −.52, *p* < .001), and a faster completion of the cleaning task arguably reflected higher cleaning motivation. An RM-ANOVA on cleaning time with odor as within-subjects variable (laundry, vanillin, air) and context as between-subjects factor (VR, 2D) yielded another significant odor × context interaction, *F*(2, 170) = 3.40, *p* = .036, η_*p*_^2^ = .04 (odor: *F*(2, 170) = 3.36, *p* = .037, η_*p*_^2^ = .04; context: *F*(1, 85) = 1.75, *p* = .189). As expected, planned contrasts showed that when laundry odor was embedded in VR, it significantly reduced total cleaning time compared with the control conditions, *F*(1, 42) = 6.55, *p* = .014, η_*p*_^2^ = .13 (laundry vs. air, *F*(1, 42) = 8.11, *p* = .007, η_*p*_^2^ = .16; laundry vs. vanillin: *F*(1, 42) = 1.16, *p* = .288, η_*p*_^2^ = .03), but not when odors were embedded in the 2D context, *F*(1, 43) = 2.05, *p* = .159 (Fig. 5b). In VR, a pleasant odor per se was not sufficient to reduce cleaning time (vanillin vs. air), *F*(1, 42) = 3.80, *p* = .058.

#### Typical hand movement frequency

Participants may adopt various cleaning strategies (e.g., circular or to and fro movements), yet they typically regress toward a dominant hand movement within the 2–9 Hz-range (movements/s). We quantified hand movement frequency mode as the dominating peak in the frequency power-spectrum histogram on the whole task, using Fourier transformation. Intuitively, a higher hand movement frequency mode (hand speed) would reflect an increased motivation to clean. An RM-ANOVA on typical hand movement frequency with factors odor (laundry, vanillin, air) and context (VR, 2D) again indicated a significant interaction effect, *F*(2, 170) = 8.97, *p* < .001, η_*p*_^2^ = .10 (odor: *F*(2, 170) = 1.10, *p* = .336; context: *F*(1, 85) = 22.51, *p* < .001, η_*p*_^2^ = .21). Planned contrasts indicated that in a VR setting, laundry odor (vs. controls) elicited higher typical hand movement frequencies on the cleaning task, *F*(1, 42) = 5.44, *p* = .025, η_*p*_^2^ = .11 (Fig. 5c) (laundry vs. air, *F* < 1; laundry vs. vanillin: *F*(1, 42) = 7.71, *p* = .008, η_*p*_^2^ = .16), whereas in a non-immersive 2D setting, typical hand movement frequency was lower, *F*(1, 43) = 5.30, *p* = .026, η_*p*_^2^ = .11. In VR, the control odor (vanillin) decreased hand speed versus odorless control (air), *F*(1, 42) = 5.33, *p* = .026, η_*p*_^2^ = .11.

#### Controlling for odor hedonics

After the main experiment, we asked participants to rate odor pleasantness (0 = “not pleasant”, 100 = “very pleasant”) and intensity (0 = “do not perceive”, 100 = “perceive very clearly”). As these scores revealed differences in perceived pleasantness (laundry odor: *Mdn* = 86, *IQR* = 73–93; vanillin: *Mdn* = 68, *IQR* = 50–83; air: *Mdn* = 50, *IQR* = 42–50) and intensity (laundry odor: *Mdn* = 91, *IQR* = 80–100; vanillin: *Mdn* = 73, *IQR* = 57–86; air: *Mdn* = 8, *IQR* = 0–31) (also see Supplementary materials) that could have impacted the odor × context interaction, these explicit hedonic factors were added as covariates to the aforementioned RM-ANOVAs; however, these factors had no influence on standardized total cleaning effort, *F*(2, 164) = 5.41, *p* = .005, η_*p*_^2^ = .06, total cleaning time, *F*(2, 164) = 3.82, *p* = .024, η_*p*_^2^ = .04, or typical hand movement frequency, *F*(2, 164) = 9.48, *p* < .001, η_*p*_^2^ = .10.

#### Summary

In sum, when participants smelled laundry odor in an immersive VR setting, this induced an increase in motivated (more energetic and faster) cleaning behavior versus exposure to the default odorless control condition (room air). As there were no differences between the pleasant odor vanillin and odorless air on motivated behavior, we argue that rather than being pleasant, odors require a semantic association with cleaning to actually drive motivated cleaning behavior in an immersive context.

### Subjective effectiveness of cleaning

Even though, unbeknownst to them, participants used three identical cleaning liquids on the cleaning task, when they were asked to identify the *most effective* cleaning liquid (one of three liquids used after laundry, vanillin, and air exposure, or none of the former), participants selected (53.1%) significantly above chance (33.3%) the liquid used after smelling laundry odor in VR, *p* = .016 (Fig. 5d).

### Odor intake

Next, we explored whether laundry odor (vs. vanillin, air) would elicit different patterns of olfactory intake (sniffing) in an immersive VR context (vs. 2D). In particular, we analyzed sniff volume (area under the curve) and speed. As volume combines amplitude and duration, these additional sniff indicators (cf. Fig. 4) are reported in the supplementary section.

#### Sniff volume

**Fig. 6:**
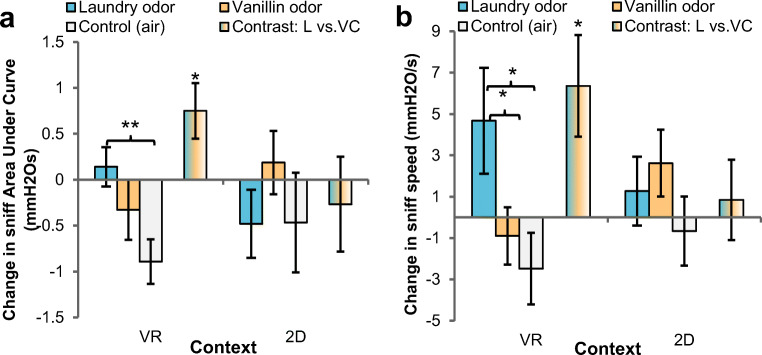
Baseline-subtracted changes in sniff volume (**a**) and speed (**b**) as combined function of odor (within subjects: laundry, vanillin, air) and context (between subjects: VR, 2D). Multicolored bars show within each context (VR, 2D) the planned contrast of laundry odor versus both control odor (vanillin) and odorless control (air). Error bars ± 1 SE. *p <.05, **p < .01

**Fig. 7:**
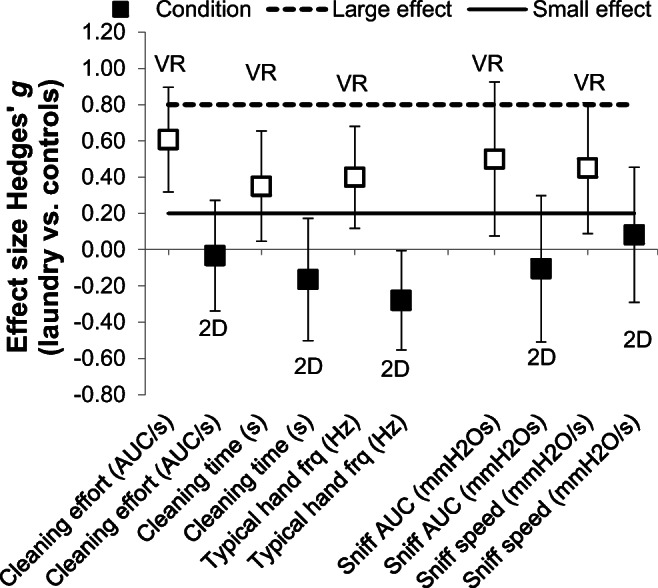
Unbiased effect sizes showing pattern of small and moderate effects on motivated cleaning behavior and air intake when laundry odor (vs. controls) was presented in VR (vs. 2D)

An RM-ANOVA on sniff volume (area under the curve: AUC) with odor (3 levels: laundry, vanillin, air) and sniff number (3 levels: 1st, 2nd, 3rd) as within-subjects variables and between-subjects variable context (2 levels: VR, 2D) yielded a significant interaction between odor and context, *F*(2, 150) = 5.25, *p* = .006, η_*p*_^2^ = .07. This interaction qualified the non-significant main effects of odor, *F*(2, 150) = 1.24, *p* = .294, and context, *F* < 1 (see Supplementary Table 1, for non-central effects of sniff number). Planned contrasts teased apart the odor × context interaction, by comparing within each context (VR, 2D) the effect of laundry odor versus both controls (vanillin, air). Notably, sniff AUC was significantly higher when laundry odor (vs. controls) was presented in VR, *F*(1, 43) = 6.12, *p* = .017, η_*p*_^2^ = .12 (laundry vs. air: *F*(1, 43) = 9.24, *p* = .004, η_*p*_^2^ = .18; laundry vs. vanillin: *F*(1, 43) = 1.71, *p* = .198), but this effect was not seen in 2D, *F* < 1 (Fig. 6a; see Fig. 7 for effect sizes). In VR, the pleasant control odor vanillin was not sufficient to induce greater sniff volume (vs. air), *F*(1, 43) = 2.69, *p* = .108.

#### Sniff speed

Higher values (mmH_2_O/s) indicate sharper and faster air intake at the start of an in-breath. An RM-ANOVA on sniff speed yielded a significant odor × context interaction, *F*(2, 150) = 3.40, *p* = .036, η_*p*_^2^ = .04 (odor: *F*(2, 150) = 2.85, *p* = .061; context: *F* < 1). Planned contrasts (odor × context) again showed that only in VR, laundry odor led to higher sniffing speed compared with both controls, *F*(1, 43) = 6.71, *p* = .013, η_*p*_^2^ = .13 (laundry vs. air: *F*(1, 43) = 5.85, *p* = .020, η_*p*_^2^ = .12; laundry vs. vanillin: *F*(1, 43) = 5.54, *p* = .023, η_*p*_^2^ = .11); but not in 2D, *F* < 1 (Fig. 6b, Fig. 7). In VR, the pleasant control odor vanillin was not sufficient to increase sniff speed (vs. air), *F* < 1.

#### Controlling for odor hedonics

The pivotal odor × context interactions could not have been driven by participants’ odor intensity and pleasantness ratings (see Supplementary results), because the RM-ANOVA outcomes did not change by adding pleasantness and intensity difference scores as covariates, sniff AUC: *F*(2, 146) = 5.93, *p* = .003, η_*p*_^2^ = .08; sniff speed: *F*(2, 146) = 4.24, *p* = .016, η_*p*_^2^ = .05. Hence, irrespective of explicit odor hedonics, sniff volume and sniff speed increased during laundry odor exposure (vs. vanillin, air) in a VR setting (vs. 2D), reflecting a greater tendency (driven by the specific laundry odor *and* VR context) to sample the olfactory environment. Thus, the semantic association with cleaning was more crucial than the odor’s pleasantness to increase air intake in an immersive context.

## Discussion

The goal of the present study was to explore the potentials of virtual reality (VR) for studying psychological and behavioral responses to odor. Because comparable studies had either been performed in sterile labs or in a field setting lacking rigorous control, researchers have been unable to dissect the contribution of test context on the link between odors and motivated behavior. Combining the strengths of these traditional methodologies, we applied VR to create a realistic, immersive, yet controlled multisensory context that was contrasted with a non-immersive, more traditional lab setting, while *olfactometry* ensured precise, computer-controlled delivery of laundry odor, vanillin, and air. This way, we could test whether motivated cleaning behavior is driven by (i) the odor’s pleasantness, (ii) the odor’s associations with washing, and—notably—whether the odor–behavior link is malleable by context.

The results clearly showed that, in the immersive VR context only, laundry odor elicited more profound olfactory intake (sniffing) (vs. vanillin, air) and faster, more energetic cleaning on a custom-designed cleaning task (vs. air). Explicit odor intensity and pleasantness (measured at the end of the experiment) were statistically controlled for, and we found no support for the hypothesis that a pleasant odor control (vanillin) drove motivated cleaning behavior (vs. odorless air) (cf. Smeets & Dijksterhuis, 2014). Because there were no differences between the pleasant odor vanillin and odorless air, we argue that a semantic association with cleaning/washing is required to induce motivated cleaning behavior. This study, with its rigorous experimental control and high ecological validity, is also the first to show that an immersive context is required for only the cleaning-associated odor to change olfactory intake and motivated behavior.

The current research supplements existing research by tapping (more deeply) into participants’ motivation to clean, by going beyond examining the mere *end result* of behavior on a cleaning task. To illustrate, Holland et al. (2005) had suggested the potential of certain odors in motivating cleaning behavior: participants exposed to a hidden cleaning product with a citrus fragrance cleaned more crumbs from a table (vs. participants in a no-scent room). In a subsequent field study, participants littered less in train wagons that were citrus-scented than in unscented wagons (de Lange et al., 2012), which again suggested the importance of a direct link between odor and behavior (supported by our data). However, rather than interpreting just the objective *end result* of cleaning, we combined video recordings with objective logging of hand movements to arrive at *online* recordings of various types of motivated behavior (e.g., faster hand movement, more invested energy).

Second, cleaning behavior was conducted up to the *subjective* criterion that participants themselves deemed the stain to be removed. Notably, objective online cleaning motivation differed mostly between laundry odor and control odor (and to a lesser extent between laundry odor and vanillin); yet, when asked to choose the most effective cleaning liquid, participants selected above chance the detergent used after smelling laundry odor in VR, even though (unbeknownst to participants) all cleaning liquids were the same. It is enhancing the subjective effectiveness of cleaning (materials) that would interest the industry concerned with adding fragrances to products, such as fast-moving consumer goods. Again, contextual factors shape the odor-driven objective motivation to clean and its subjectively perceived effectiveness.

Our findings also expand a body of literature that has underlined the importance of context in shaping *sensory perception*. The sensory perception of odors can be modulated by the behavioral act of “sniffing” (i.e., the nasal equivalent of eye tracking), controlled by a dedicated sensorimotor subsystem (Mainland & Sobel, 2006). Our aim with sniffing (the nasal equivalent of eye tracking) was to have an online indicator of odor intake (cf. prior research, which did not record sniffs: de Lange et al., 2012; Holland et al., 2005), to be able to observe changes in participants’ olfactory intake patterns as a function of odor and context. Prior research has shown that expressing fear (Susskind et al., 2008) and smelling fear odor (de Groot et al., 2012) *increases* sensory intake volume through the nose; yet, the present research filled a knowledge gap by showing that an immersive context and congruent laundry odor “turned on” the odor intake apparatus, by increasing sniff volume and speed. Hence, this is the first study to show that not only motivated cleaning behavior, but also the olfactomotor system is tuned differently to the *same odors* in a different context. These findings confirm that the olfactory percept is more than pure raw sensory data: like other modalities, it is subject to top-down control by means of active cortical processing (see Gilbert & Sigman, 2007) that weighs in (immersive) contextual information.

The present study attempted to create a multimodal, lively virtual test environment for sensory evaluation of odors, and to compare its effect with a traditional context-poor 2D setting.

To our knowledge, this is the first study with an olfactory display in VR that released odors through interaction with the participant in the virtual world (i.e., a button press opened the washing machine and simultaneously caused odor release); it required accurate timing of odor delivery and fixed-concentration airflow. Our data showed that realism and immersion of the VR scenario were excellent, with sound contributing highly to perceived immersion, and odors and visuals being close followers. Because there were various elements that set apart our VR setting from the 2D one (i.e., sounds, touch, neatly integrated smells, interaction, movement), the question for future research is to further dissect the contributions of each of these elements to our main findings. To reiterate, compared with a “context-deprived” setting showing a 2D image of the VR scenario on a regular monitor, olfactory sampling and cleaning behavior were found to be enhanced when a congruent laundry odor was administered in VR.

### Constraints on generality

The obtained insight that odor and context interact to shape motivated cleaning behavior was limited to a female test sample, which imposes a constraint on generality (Simons, Shoda, & Lindsay, 2017). Only female participants were recruited for this proof-of-principle study, because females generally have a better sense of smell (Sorokowski et al., 2019), thus increasing this study’s potential for effectiveness in terms of finding interactions between odor and context. Currently, we do not have evidence that our findings generalize to males. However, we have no reason to believe that cleaning-related odor would *not* elicit motivated behavior in males (cf. Holland et al., 2005, who had minorities of males in their samples using citrus smell), because (i) gender differences in smell abilities yield only small effect sizes, and (ii) we expect cleaning behavior to be triggered by representations of cleaning behavior that are activated by laundry odor through mere association, and males are expected to have these associations as well. It could be, however, that individuals who wash more frequently display a stronger motivation to clean because of more accessible representations of cleaning behavior activated by laundry odor. Our data showed that 86% of the female test sample washed their clothes multiple times a month. These representations could be even more accessible and strong for individuals doing the laundry manually, which is not typical in the Western world with washing machines being widespread, which is another constraint on generality: the test sample being Western, Educated, Industrialized, Rich, Democratic (WEIRD) (Henrich, Heine, & Norenzayan, 2010). Arguably, motivation to engage in cleaning behavior is further influenced by cultural norms and values, and future research could identify and introduce these factors as moderators to our design, adding an additional layer of context to the experiment.

### Theory

Our findings thus show that motivated goal-directed behavior as induced by odors is malleable by context. Based on related research in which a cleaning-related odor (citrus) increased cleaning behavior, both in the lab (Holland et al., 2005) *and* in the field (de Lange et al., 2012), it had been inviting to conceptualize this form of odor “priming” as a *reflex-like* stimulus–response phenomenon (cf. Carpenter, 1874; James, 1890), with prototypical (cleaning) behavior *always* following (cleaning-related) odor exposure. However, such a perspective—frequently found in other domains of olfactory research as well (e.g., social communication; for a discussion, see de Groot et al., 2017)—neglects the top-down mediating role that cognitions (influenced by context) have on the link between perception and action. As we have observed here, this perception–action link is not (always) static.

Instead, our findings seem best intelligible from situated cognition theory, from which odors are expected to evoke goal-directed behavior *particularly* when the current situation matches a stored “conceptualization” of a similar situation (e.g., Barsalou, 2009; Barsalou, Niedenthal, Barbey, & Ruppert, 2003). From a Bayesian perspective, cleaning-related situated conceptualizations become accessible when the present situation (e.g., laundry odor *and* realistic multisensory VR washing setting) forms a “good match” (likelihood) with the original situation. In turn, these activated situated conceptualizations (SC) produce pattern completion inferences that are implemented as multimodal simulations (Barsalou, 2016a), which could explain enhanced sniffing and motivated cleaning behavior emerging especially (and most consistently) when participants smelled laundry odor in VR. In sum, the present research shows that humans are no “odor zombies” that react to the same odor the same way across situations; rather, the context at hand determines whether odors induce *motivated behavior*, a novel insight that carries several practical implications.

### Practice

The insights from this research suggest marked changes in traditional sensory testing practices used in industry, which have relied on quick, low-cost testing of fragrances in sterile booths. Here, we applied VR to create a test context that decisively influenced the way fragrances motivate human behavior and perception (vs. a non-immersive sterile setting). We suggest combining VR to create an immersive test context with an olfactometer that can release several fragrances (serially or in parallel) in a relatively short span of time. Furthermore, the custom-designed cleaning task and sniff recordings we used here allowed for going beyond asking consumers about their experience, and participants remained unaware of what was being tested and why, which enhanced experimental validity because odors generally affect us on an implicit level (e.g., Degel & Köster, 1999). We suspect that the merit of applying VR to odor-based research extends from fragrances added to certain products (e.g., laundry odor) to other domains in which odors are important, including eating behavior (foods and drinks) and social communication (e.g., deodorants).

The downside of taking into account all of the above in product testing is that it adds time, cost, and complexity compared with the tradition of testing fragrance liking in a laboratory setting, although revenues will likely increase. Fragrances are among the most important consumer benefits for a wide variety of products (including laundry detergent), and fragrances also form a large part of the cost of the product, making it of utmost importance to test odors and their effects on consumers in the most efficient way possible, under the right settings. Admittedly, significant methodological challenges related to odor “production” need to be solved before olfactory displays can be introduced as a mainstream technology (Salminen et al., 2018); nevertheless, there are affordable and easy-to-implement methods for adapting VR technology to sensory evaluation, without prohibitive amounts of expensive equipment or programming knowledge (Stelick, Penano, Riak, & Dando, 2018). For instance, a successful virtual environment was formed by showing 360**°** videos overlaid with audio, text, and images to simulate a typical sensory evaluation ballot within the VR headset (Stelick et al., 2018). Bangcuyo et al. (2015) created a “virtual coffee house” environment using a wall of nine 48-inch high-definition LCD screens with video and audio recordings from a local coffee shop along with cinnamon aroma. In line with our research, consumer preferences for coffee changed from standard sensory booths to the virtual coffee house (Bangcuyo et al., 2015; also see Andersen, Kraus, Ritz, & Bredie, 2019). Hence, many benefits can be taken from immersive technologies such as VR in different parts of the business.

## Conclusion

Our results form a proof of concept that immersive virtual reality (VR) has a crucial impact on participants’ perception of odors and concomitant motivated behavior. Compared with a non-immersive setting deprived of multisensory stimulation (i.e., the sterile lab), VR provided us with a tool to create a highly controlled testing environment, with its realistic multisensory features imbuing odors with their real-world relevance and motivating properties. The application of this VR framework can easily be modified to create more affordable immersive and dynamic contexts in future testing settings, as per our recommendations. The present findings may prove useful in sensory and consumer testing across the whole spectrum of the chemical senses, from fragrances to flavors of foods.

## Electronic supplementary material

ESM 1 (PDF 220 kb)
