# Supplementary material for: From sterile labs to rich VR: Immersive multisensory context critical for odors to induce motivated cleaning behavior
Source: Behav Res Methods. 2020 Jan 21;52(4):1657–70. doi: 10.3758/s13428-019-01341-y (PMC7406481; doi:10.3758/s13428-019-01341-y)
Supplement: Supplementary file 1 — (PDF 220 kb) [file 13428_2019_1341_MOESM1_ESM.pdf]

Supplementary results

Manipulation checks

Odor. The results of the current study (VAS scores, 0 = "not al all"; 100 = "very much") confirmed pilot-test results, showing that regardless of context ($F < 1$), vanillin (and not laundry odor) was perceived as strongly food-related, $F(1, 86) = 161.88$, $p < .001$, $\eta_p^2 = .65$ (e.g., baking cake: $M_{\text{vanillin}} = 55.59$, $SD = 32.85$; $M_{\text{laundry}} = 9.25$, $SD = 16.68$), whereas laundry odor (and not vanillin) was perceived as strongly related to laundry activities, $F(1, 86) = 484.40$, $p < .001$, $\eta_p^2 = .85$ ($M_{\text{laundry}} = 88.61$, $SD = 14.92$; $M_{\text{vanillin}} = 21.20$, $SD = 23.04$).

Although concentrations of laundry odor and vanillin were pilot-tested to be equally pleasant and intense, we subjected participants' actually perceived odor intensity and pleasantness ratings to a repeated measures (RM) ANOVA with odor (3 levels: laundry, vanillin, air) as within-subjects factor and context (2 levels: VR, 2D) as between-subjects factor. This test yielded a significant effect of odor on both intensity, $F(2, 172) = 300.72$, $p < .001$, $\eta_p^2 = .78$, and pleasantness, $F(2, 172) = 92.73$, $p < .001$, $\eta_p^2 = .52$. Follow-up contrasts showed a significant linear increase in pleasantness and intensity (air<vanillin<laundry), in *both* VR and 2D, intensity-VR: $F(1, 42) = 220.72$, $p < .001$, $\eta_p^2 = .84$; pleasantness-VR: $F(1, 43) = 121.81$, $p < .001$, $\eta_p^2 = .74$; intensity-2D: $F(1, 44) = 410.72$, $p < .001$, $\eta_p^2 = .90$; pleasantness-2D: $F(1, 43) = 104.70$, $p < .001$, $\eta_p^2 = .71$. As can be seen in the main text, intensity and pleasantness difference scores were calculated and added as covariates into the main analyses, but to no effect.

VR. All participants in the VR condition filled out self-report questions regarding their experience of the VR-environment. Based on scores that were generally on the high end of 7-point Likert scales (1: "not at all"; 7: "very much"), participants had strong feelings of realism and immersion. For instance, the VR experience strongly matched the real world ($M = 5.57$, *SD*

= .93), and both sounds (*M* = 6.18, *SD* = .69), odors (*M* = 5.75, *SD* = 1.01), and images (*M* = 5.36, *SD* = 1.18) made the VR scenario seem real.

Perceived t-shirt cleanliness (VR, 2D). We also analyzed participants' visual evaluation (per odor condition: laundry, vanillin, air) of the cleanliness of a t-shirt that was *shown* in either a VR setting or 2D context (hence, these are not ratings of the cloth they cleaned during the cleaning task). Answers on five questions were recorded on visual analog scales (VAS: 0-100).

To assess the impact of odor (within-subjects: laundry, vanillin, air) and context (between-subjects: VR, 2D) on the question "How clean is this t-shirt?", a RM-ANOVA was conducted, which only showed a significant effect of context, $F(1, 88) = 9.86$, $p = .002$, $\eta_p^2 = .10$ (odor: $F(2, 176) = 2.84$, $p = .061$; odor x context: $F(2, 176) = 2.36$, $p = .097$).

Similar effects were obtained for "How dirty is this t-shirt?", context: $F(1, 88) = 7.44$, $p = .008$, $\eta_p^2 = .08$ (odor: $F(2, 176) = 1.91$, $p = .152$; odor x context: $F < 1$), "How white is this t-shirt?", context: $F(1, 88) = 10.15$, $p = .002$, $\eta_p^2 = .10$ (odor: $F(2, 176) = 1.79$, $p = .169$; odor x context: $F(2, 176) = 2.16$, $p = .118$), "How visible is the stain – if you see any?", context: $F(1, 88) = 3.24$, $p = .075$ (odor: $F(2, 176) = 1.78$, $p = .171$; odor x context: $F(2, 176) = 3.09$, $p = .048$), and "How content are you with the laundry result?", context: $F(1, 88) = 9.32$, $p = .003$, $\eta_p^2 = .10$ (odor: $F(2, 176) = 2.32$, $p = .102$; odor x context: $F < 1$).

A coherent pattern emerged. Compared to the VR condition, participants in the 2D setting perceived the t-shirt shown there to be cleaner, whiter, and less dirty, they had more trouble seeing the stain on that t-shirt, and were more satisfied with the laundry result. Arguably, the (visual features of the) VR setting generally engaged participants to evaluate the t-shirt more critically. Since there was neither an effect of odor, nor of odor x context, the key results

reported in the main manuscript are unlikely to have been driven by these purely context-specific differences in perceived t-shirt cleanliness.

Washing frequency. At the end of the experiment, we asked participants about their frequency of doing the laundry (manually vs. machine). Non-parametric tests indicated that subjects in VR and 2D had no significant differences in washing frequency (*Z*s < 1). Hence, the main results (cf. main text) cannot be due to a priori differences in washing habits (Figure S1).

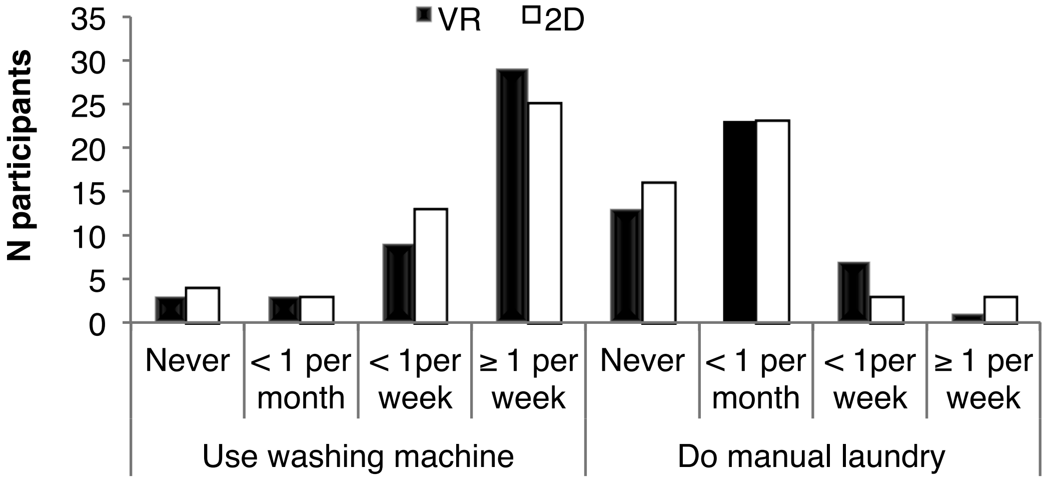

Figure S1. Frequency of washing behavior split by the test context (VR, 2D).

Sniffing behavior

Sniff amplitude. A RM-ANOVA on sniff amplitude with odor and sniff number (within-subjects) and context (between-subjects) as factors revealed a significant odor x context interaction, $F(2, 150) = 5.77, p = .004, \eta_p^2 = .07$, which qualified the non-significant effects of odor, $F(2, 150) = 1.70, p = .187$, and context, $F < 1$. Planned contrasts corroborated that in VR, laundry odor induced greater sniff amplitudes versus both controls, $F(1 ,43) = 11.03, p = .002$, $\eta_p^2 = .20$ (laundry vs. air: $F(1, 43) = 10.91, p = .002, \eta_p^2 = .20$; laundry vs. vanillin: $F(1, 43) = 6.62, p = .014, \eta_p^2 = .13$), whereas no effect was found in 2D, $F(1, 43) = 1.55, p = .220$ (Figure

S2). In VR, the pleasant control odor vanillin was not sufficient to increase sniff amplitude (vs. air), $F(1, 43) = 1.78, p = .189$.

Sniff duration. A RM-ANOVA on sniff duration, however, yielded neither a significant odor x context interaction, $F(2,150) = 2.06, p = .131$, nor effects of odor, $F < 1$, and context, $F < 1$. There were no significant differences in sniff duration for laundry odor vs. controls in VR ($F(1, 43) = 1.21, p = .278$) and 2D ($F < 1$) (Figure S2).

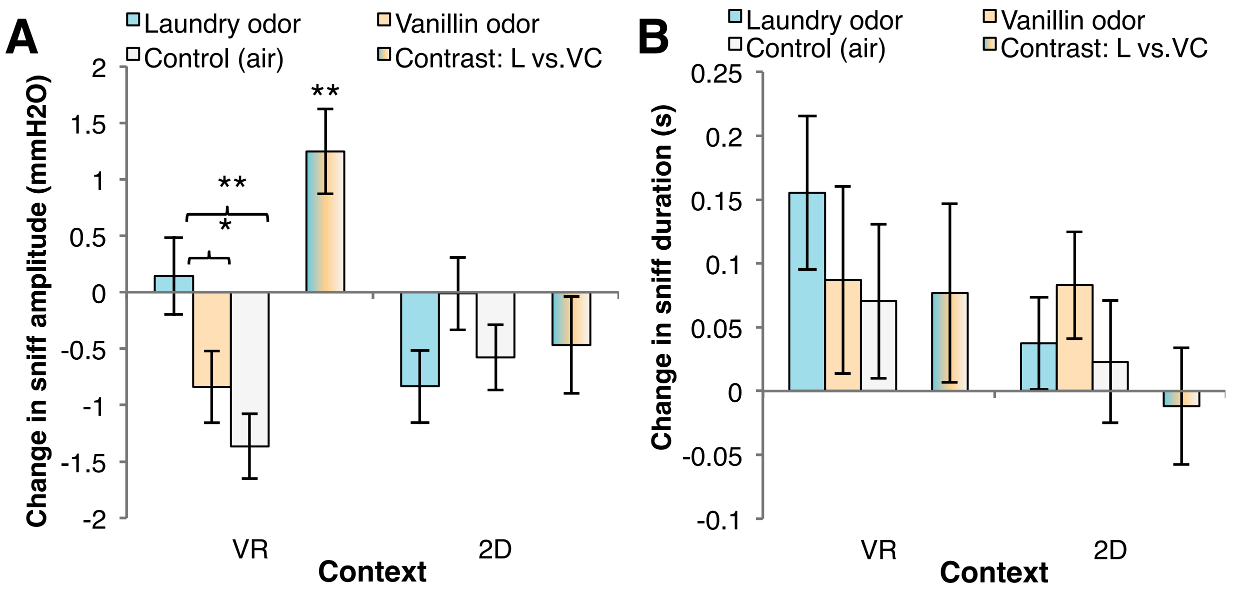

Figure S2. Baseline-subtracted changes in sniff amplitude (**A**) and duration (**B**) as combined function of odor (within-subjects: laundry, vanillin, air) and context (between-subjects: VR, 2D). Multi-colored bars show within each context (VR, 2D) the planned contrast of laundry odor versus both control odor (vanillin) and odorless control (air). Error bars ± 1 SE. *p <.05, **p < .01.

Controlling for odor hedonics. The pivotal odor x context interactions could not be driven by participants' odor intensity and pleasantness ratings, because the RM-ANOVA outcomes did not change by adding pleasantness and intensity difference scores as covariates, sniff amplitude: $F(2, 146) = 6.08, p = .003, \eta_p^2 = .08$; sniff duration: $F(2, 146) = 1.66, p = .193$.

Sniff speed. Sniff number significantly affected sniff speed, $F(2, 150) = 6.09$, $p = .004$, $\eta_p^2 = .08$, with a higher velocity of air intake on the second sniff ($M = 4.59$, $SD = 14.70$) versus the first ($M = -.89$, $SD = 8.94$), $p = .008$, and third sniff ($M = -.14$, $SD = 11.30$), $p = .002$ (cf. Table S1).

Sniff time-to-top. For purely exploratory purposes, we examined the time at which the maximum amplitude of the sniff would be reached, called sniff time-to-top. A repeated measures ANOVA on sniff time-to-top with odor (3 levels: laundry, vanillin, air) and sniff number (3 levels: 1st, 2nd, 3rd) as within-subjects variables and context (2 levels: VR, 2D) as between-subjects variable showed a significant interaction between odor and context, $F(2, 150) = 3.42$, $p = .035$, $\eta_p^2 = .04$. This interaction qualified a non-significant main effect of odor, $F(2, 150) = 2.57$, $p = .080$, and a significant main effect of context, $F(1, 75) = 6.57$, $p = .012$, $\eta_p^2 = .08$. The remaining effects were not significant (sniff number: $F(2, 150) = 2.26$ $p = .110$; sniff number x context: $F(2, 150) = 1.54$, $p = .218$; odor x sniff number: $F(4, 300) = 1.47$, $p = .210$; odor x sniff x context: $F(4, 300) = 1.78$, $p = .132$).

Planned contrasts following up on the relevant odor x context interaction showed a significant slower time-to-top when the laundry odor (vs. control odor, odorless control) was situated in the VR context, $F(1, 43) = 4.54$, $p = .039$, $\eta_p^2 = .09$ (laundry: $M = 5.88$, $SD = 11.15$; vanillin: $M = .21$, $SD = 12.03$; air: $M = 2.41$, $SD = 12.32$), but not when laundry odor was presented in a 2D setting, $F < 1$ (laundry: $M = 5.82$, $SD = 11.04$; vanillin: $M = 6.52$, $SD = 11.78$; air: $M = 3.70$, $SD = 11.27$). In the VR setting, the difference between laundry odor and odorless control on sniff time-to-top was not significant, $F(1, 43) = 2.12$, $p = .152$; whereas significant differences were encountered for the laundry-vanillin comparison, $F(1, 43) = 5.60$, $p = .023$, $\eta_p^2 = .12$.

Motivation to clean

We further explored a participant's motivation to clean as a function of odor and context using the following parameters: average number of cleaning blocks, strategy diversity, average cleaning frequency, and non-time-standardized measures of total cleaning effort: area under the curve (AUC), and power root mean squared (RMS).

Average number of cleaning blocks. First, a RM-ANOVA was conducted on the average number of cleaning blocks with odor (3 levels: laundry, vanillin, air) as within-subjects factor and context (2 levels: VR, 2D) as between-subjects factor. This analysis neither yielded effects of odor, $F < 1$, and context, $F < 1$, nor a significant interaction, $F < 1$ (Figure S3A).

Strategy diversity. The same test on "strategy diversity", which was essentially the spread of cleaning frequencies, yielded all Fs < 1 (Figure S3B).

Average cleaning frequency. The auto power spectrum also allowed for calculating the average cleaning frequency (i.e., the weighted average of cleaning frequencies). Another repeated measures ANOVA on average cleaning frequency (Hz) with the same factors yielded only a significant effect of context, $F(1, 85) = 5.27$, $p = .024$, $\eta_p^2 = .06$. Participants in the VR context had a higher average cleaning frequency than participants in the 2D context (Figure S3C). Importantly, there was no significant interaction between odor and context, $F < 1$, and no significant main effect of odor, $F < 1$.

Total cleaning effort (area under the curve). An arguably intuitive measure of total cleaning effort was inspected by looking at the area under the curve (AUC) in the acceleration x time domain (hand speed: m/s). A repeated measures ANOVA on AUC with factors odor (within-subjects) and context (between-subjects) showed a non-significant interaction between

odor and context, $F(2, 170) = 1.38$, $p = .255$. There were also no main effects of odor, $F(2, 170) = 1.64$, $p = .197$, and context, $F < 1$ (Figure S3D).

Total cleaning effort (power RMS). Although AUC is more straightforward to interpret, power RMS is a routinely used (and arguably better) outcome measurement of total effort in power spectral analysis. Per condition, the RMS (root mean square) of the Fourier-transformed auto power spectrum was calculated (µgrams^2). To test whether total cleaning effort is higher when the laundry odor prime was embedded in the VR scenario, a repeated measures ANOVA was carried out with odor (within-subjects) and context (between-subjects). Again, all effects were non-significant: odor x context, $F(2, 170) = 2.94$, $p = .055$; odor: $F(2, 170) = 1.37$, $p = .257$; context: $F(1,85) = 1.11$, $p = .295$.

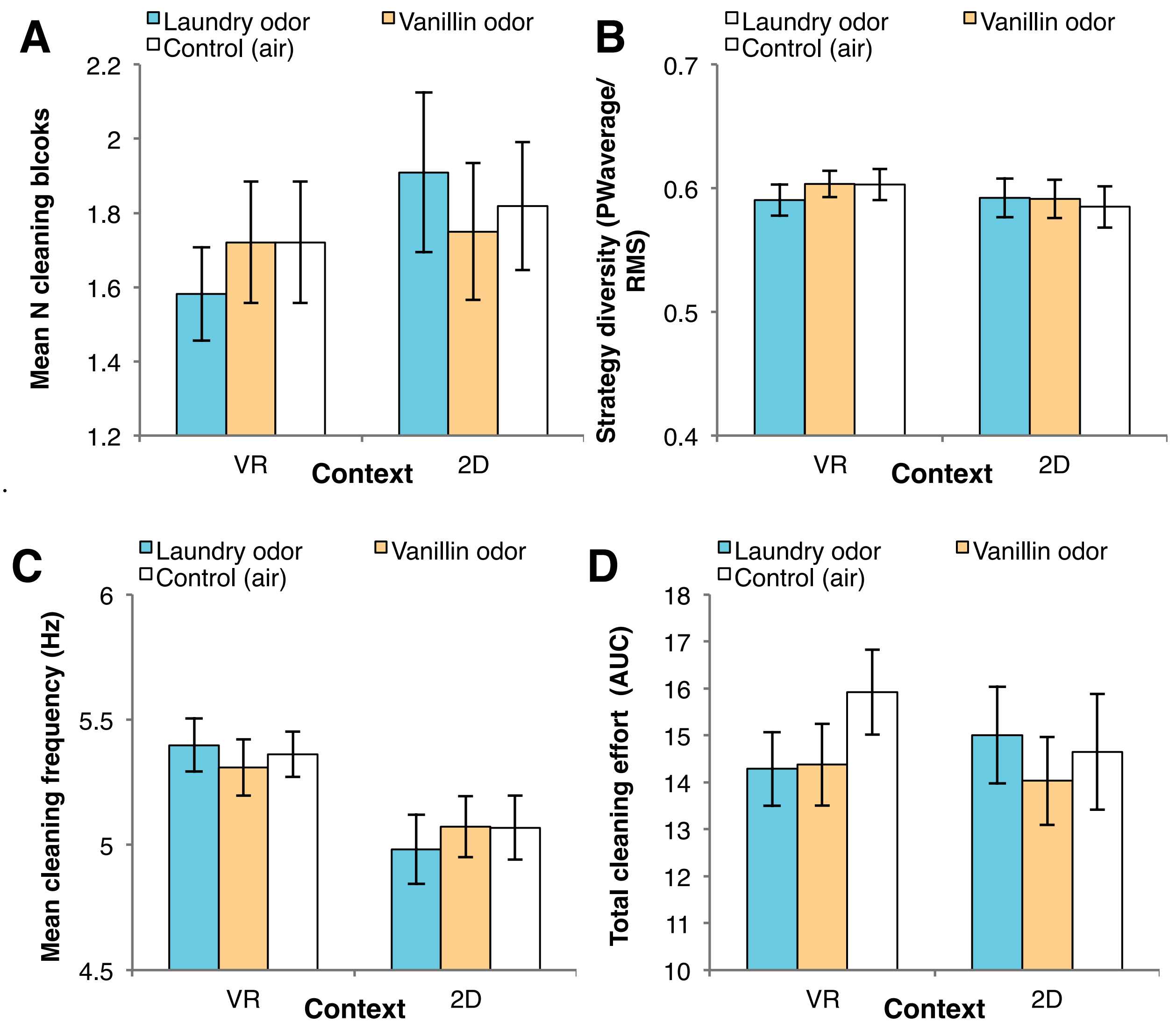

Figure S3. Cleaning behavior as a function of odor (laundry, vanillin, air) and context (virtual reality: VR, two-dimensional: 2D). (**A**) Mean number of cleaning blocks. (**B**) Mean strategy diversity. (**C**) Average cleaning frequency (Hz). (**D**) Total cleaning effort (area under the curve).

Supplementary Table 1

Effects of sniff number on sniff parameters

**Sniff variable**	Sniff number: $F(2, 150)$	Context x Sniff number: $F(2, 150)$	Odor x Sniff number: $F(4, 300)$	Context x Odor x Sniff number: $F(4, 300)$
Volume	$F < 1$	$F < 1$	**F=3.67, p=.006**	F=1.01, p=.401
Amplitude	$F < 1$	**F=3.17, p=.045**	$F < 1$	$F < 1$
Duration	$F < 1$	$F < 1$	**F=6.63, p<.001**	$F < 1$
Speed	**F=6.09, p=.004**	F=2.21, p=.114	F=1.99, p=.096	F=1.28, p=.279

Note Significant results in bold.

Cross-instrument relations

For exploratory purposes, we calculated correlations between the various sniffing behavior, cleaning task, and perceptual judgment variables. However, the lack of precise temporal synchrony in the recording of these data has arguably rendered finding significant correlations unlikely. Indeed, we were unable to perceive significant correlations across measures (*p*s $> .05$); yet, we presume based on our data and theorizing that such relations are likely to be found with more temporal synchrony in measurement. Even though correlations were absent, the pattern of results on the sniffing and cleaning task variables coherently showed that participants displayed increased olfactory simulations and cleaning behavior when they were primed with laundry odor in an enriched VR context only.
